# Association among serum and salivary A. actinomycetemcomitans specific immunoglobulin antibodies and periodontitis

**DOI:** 10.1186/s12903-020-01258-5

**Published:** 2020-10-15

**Authors:** Gaetano Isola, Alessandro Polizzi, Romeo Patini, Sebastiano Ferlito, Angela Alibrandi, Giuseppe Palazzo

**Affiliations:** 1 Department of General Surgery and Surgical-Medical Specialties, School of Dentistry, University of Catania, Via S. Sofia 78, 95124 Catania, Italy; 2 Fondazione Policlinico Universitario A. Gemelli IRCCS, Institute of Dentistry and Maxillofacial Surgery, Università Cattolica del Sacro Cuore, 00168 Rome, Italy; 3 Department of Economical, Business and Environmental Sciences and Quantitative Methods, University of Messina, Messina, Italy

## Abstract

**Background:**

The aim of this study was to assess the association between serum and salivary Immunoglobulin (Ig) *Aggregatibacter actinomycetemcomitans* (*A. actinomycetemcomitans)* specific antibodies in healthy controls (HC) and periodontitis (PT) patients. Furthermore, the objectives were to determine whether PT influenced serum *A. actinomycetemcomitans* specific antibodies and whether serum or salivary antibodies against *A. actinomycetemcomitans* IgG were mediated by serum high-sensitivity c-reactive protein (hs-CRP).

**Methods:**

Fifty-three patients with periodontitis and 48 HC were enrolled in the present study. Patients were regularly examined and characterized by clinical, salivary and blood samples analyses. *A. actinomycetemcomitans* IgA and IgG antibodies and hs-CRP were evaluated using a commercially available kit. The Spearman Correlation Test and Jonckheere-Terpstra Test were applied in order to assess the interdependence between serum *A. actinomycetemcomitans* IgG antibodies and clinical periodontal parameters. To evaluate the dependence of the serum and salivary *A. actinomycetemcomitans* IgG levels from possible confounders, univariate and multivariable linear regression analyses were performed.

**Results:**

Compared to HC, patients with PT had significantly higher IgA [serum: PT, 1.89 (1.2–2.2) EU vs HC, 1.37 (0.9–1.8) EU (*p* = 0.022); saliva: PT, 1.67 (1.4–2.1) EU vs HC, 1.42 (0.9–1.6) EU (*p* = 0.019)] and *A. actinomycetemcomitans* IgG levels [serum: PT, 2.96 (2.1–3.7) EU vs HC, 2.18 (1.8–2.1) EU (*p* < 0.001); saliva, PT, 2.19 (1.8–2.5) EU vs HC, 1.84 (1.4–2) EU (*p* = 0.028)]. In PT patients, serum *A. actinomycetemcomitans* IgG were associated with a proportional extent of PT and tooth loss (P-trend value< 0.001). The univariate regression analysis demonstrated that PT (*p* = 0.013) and high hs-CRP (*p* < 0.001) had a significant negative effect on serum and salivary *A. actinomycetemcomitans* IgG levels. The multivariate regression analysis showed that PT (*p* = 0.033), hs-CRP (*p* = 0.014) and BMI (*p* = 0.017) were significant negative predictors of serum *A. actinomycetemcomitans* IgG while hs-CRP (p < 0.001) and BMI (*P* = 0.025) were significant negative predictors of salivary *A. actinomycetemcomitans* IgG.

**Conclusions:**

PT patients presented a significantly higher serum and salivary *A. actinomycetemcomitans* IgA and IgG compared to HC. There was a significant increase in serum *A. actinomycetemcomitans* IgG when patients presented a progressive extent of PT. Moreover, PT and hs-CRP were significant negative predictors of increased salivary and serum *A. actinomycetemcomitans* IgG levels.

**Trial registration:**

The study was retrospectively registered at clinicaltrials.gov (NCT04417322).

## Background

Personalized dentistry is an ongoing multidisciplinary model which is aimed at tailoring healthcare with dental research, practice and decisions specifically customized for the single patient [1]. In this model, diagnostic tests are usually applied for the approach to periodontitis (PT) for selecting optimal and appropriate therapies on the specific patient’s context through a genetic subject or other physiological, epidemiological, or molecular analyses [2].

PT is a chronic inflammatory disease correlated with a plethora of systemic disorders such as endothelial dysfunction [3], diabetes [4], and metabolic syndrome [5]. PT is mainly caused by pathogenic pathogens organized in biofilm which, due to the host inflammatory response, results in periodontal tissues destruction, bone resorption and finally in tooth loss [6].

Of the main periodontal pathogens, during the last few decades, an important role in the aetiology and progression of PT has been shown mainly by certain bacteria, such as *Tannerella forsythia*, *Porphyromonas gingivalis* (*P. gingivalis*), and *Aggregatibacter actinomycetemcomitans* (*A. actinomycetemcomitans*) [7, 8]. The presence of these bacteria has been demonstrated, in periodontal tissues, to stimulate the first innate host tissue response against the release of host inflammatory mediators from the junctional epithelium and from the fibroblasts, macrophages and neutrophils activated by lymphocytes which in term, could finally lead to periodontal tissue destruction and tooth loss [9, 10].

*A. actinomycetemcomitans* is a facultative Gram-negative bacterium considered to have a key role in PT development due to its important orchestration of the gingival dysbiosis during the early stages of disease [11]. Several reports have highlighted high serum immunoglobulin (Ig) -A and IgG antibodies levels against *A. actinomycetemcomitans* in the early stages of some diseases, such as cardiovascular diseases [12], diabetes [13] and rheumatoid arthritis [14]. For these reasons, it is conceivable to find biomarkers which could help to understand the aetiology of PT better and to prevent risk-related factors associated with PT.

Moreover, some cohort studies have highlighted the strict role of periodontal bacteria and a positive association among *A. actinomycetemcomitans* antibody levels and coronary artery calcification [13]. All of these studies have shown that the exclusive presence of some pathogens such as *A. actinomycetemcomitans* and *P. gingivalis* at gingival level represents the main determinant of a systemic antibody response by the host, suggesting that serology may mark the history of past and present periodontal infection [15]. Some reports have shown that serum antibody levels against pathogenic periodontal bacteria remain stable over time [15], while they decrease only transiently after the treatment of PT [16], with significant a significant association with the progression of PT [17].

Based on these findings, this study was aimed at further analyzing the relationship between serum Ig antibodies against *A. actinomycetemcomitans* antibodies and PT and to explore the main predictors that can influence gingival health in PT and healthy subjects. Additionally, the purposes were 1) to evaluate the impact of PT on the serum and salivary IgG antibodies against *A. actinomycetemcomitans*; 2) whether there was an association between serum and salivary IgG antibodies against *A. actinomycetemcomitans*; 3) whether hs-CRP had a significant influence on serum and salivary IgG antibodies against *A. actinomycetemcomitans*.

## Methods

### Study design

A number of 221 subjects were enrolled from May 2016 to December 2019 at the School of Dentistry of the University of Messina, Messina, Italy. The local International Review Board (IRB) approved the protocol of the study prior to the patient’s enrolment (#12–16). The trial was registered at clinicaltrials.gov (NCT04417322). All enrolled subjects were carefully instructed on the study characteristics and who then gave their written informed consent. The study was performed in accordance with the Declaration of the World Medical Association 1975 in Helsinki guidelines, revised in 2000 and followed the STROBE (Strengthening The Reporting Of Observational Studies In Epidemiology) guidelines (Additional file 1).

Group of patients were selected on sex and on a specific age range (from 35 to 65 years old) in order to obtain a similar proportion of cases on categories, sex and age, defined by the selection variable. A total of 48% of patients were males ranging in age from 41 to 57 years old.

Sociodemographic parameters such as gender, age, and a complete medical history and medications were recorded at baseline in all enrolled subjects by the same calibrated clinician. Subsequently, each patient underwent an oral assessment and a periodontal charting by the recording, at six sites per tooth by means of standard periodontal parameters [18] using a manual periodontal probe (PCP-15; Hu-Friedy, Milan, Italy).

The diagnosis of PT was performed, as 1) having a number of teeth ≥16; 2) having at least 40% of periodontal sites with bleeding on probing (BOP), a probing depth (PD) ≥ 4 mm and clinical attachment level (CAL) ≥ 2 mm [6]; 3) having ≥2 mm of interdental alveolar bone loss verified through Rinn radiographs [3].

Healthy subjects, matched for gender and age, had no systemic disease, did not take any drugs, and presented good oral health and had no sites with PD ≥ 4 mm or CAL ≥ 4 mm and there X-rays did not show any signs of bone loss; consequently, these patients were enrolled as healthy control group.

The exclusion criteria for all patients were: (1) consumption of contraceptive drugs; (2) consumption of immunosuppressive, antibiotics or anti-inflammatory at least the previous 3-months preceding the study; (3) status of pregnancy or lactation; (4) history of drinking; (5) allergy episodes to drugs or local anaesthetics; (6) consumption of any drugs that could give gingival hyperplasia.

### Study population

**Fig. 1:**
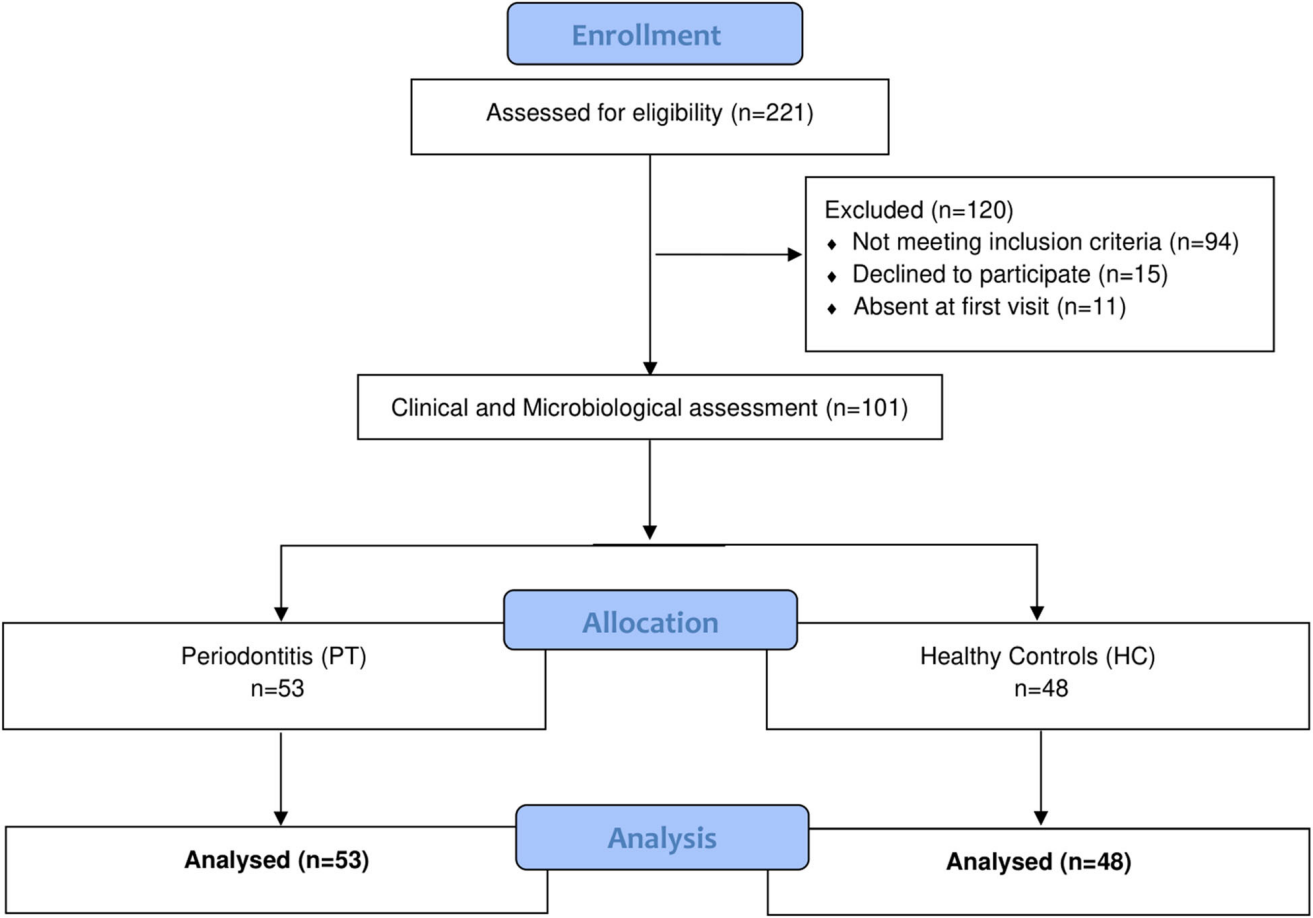
Flowchart of the study

The present trial was designed as a cross-sectional study. After a preliminary screening, 120 subjects were initially excluded because they did not present all inclusion criteria (*n* = 94), declined to participate (*n* = 15) or were absent at the first visit (*n* = 11). Finally, a number of 53 patients with PT and 48 healthy subjects were analyzed (Fig. 1).

### Data collection

At the first visit, the sociodemographic characteristics of the enrolled patients such as age, gender, body mass index (BMI), anamnesis and medications were recorded. BMI was valued by calculating the patient’s weight and height. Diagnosis of diabetes mellitus was determined, at this stage, with the presence of a fasting blood glucose ≥7 mmol/l. The presence of any oral disorder, if present, was recorded during this phase.

The oral assessment included, at six sites per tooth, the recording of clinical periodontal indexes such as PD, CAL, BOP and plaque score (PI) [19]. BOP was registered by the appearance of gingival bleeding after probing up to 30 s during PD assessment. CAL was documented as PD plus the presence of gingival recession, using the cementoenamel junction as reference.

The inter-examiner reliability test was performed calculating the intraclass correlation coefficient (ICC) and demonstrated a good degree of reliability for CAL (ICC = 0.824). The intra-examiner reliability was performed, for the principal and control examiners, on 6 patients randomly chosen per group. The variable CAL recorded resulted in a good agreement both for the principal (ICC = 0.827) and control (ICC = 0.829) examiner.

### Laboratory analyses

During the first examination, the serum and saliva sampling was performed on each patient by the same examiner between 8.00 and 9.00 am (A.P.). Each patient was asked to avoid eating, drinking and brushing their teeth during the 12 h preceding plasma and saliva sampling. For the collection of saliva, cotton rolls were used which were chewed for two minutes by the patient using a specific kit (Salivette®, Sarsted, Verona, Italy). Immediately after, the salivary and serum samples were centrifuged at 4 °C (1000 xg for 2 min). After sampling, blood samples were stored at − 80 °C, and saliva samples at − 20 °C. In all patients, all analyses were achieved at the same centre after overnight fasting. Glucose, fibrinogen, and plasma lipids levels were obtained by routine laboratory analysis. The hs-CRP was measured using a nephelometric assay kit and expressed in milligrams per decilitres (mg/dl).

All serum and saliva samples were diluted with PBS to a 1:100 ratio as previously shown [20]. For serum and saliva samples for IgG antibodies, 100 μl of each diluted serum and saliva sample was added to individual well and incubated for 1 h at room temperature. 100 μl of IgG conjugate was added to each well and incubated at room temperature for 1 h (Goat Antihuman IgG HRP 1:5000 concentration using a blocking buffer) using ELISA kit (R&D Systems, Minneapolis, MN, and Sigma-Aldrich, St Louis, MO). The lower limit of the assay was 0.005 ng/mL. 100 μl of sulphuric acid (H_2_SO_4_) stop solution was then added to stop the reaction and the optical density was taken at 450 nm into iMARK microplate reader. Based on optical density, values were calculated for serum and salivary IgG antibodies (iMARK microplate reader, BIORAD, Hercules, California). For serum and saliva samples for IgA antibodies, samples were diluted with PBS to 1:100 (serum) and 1:50 (saliva) ratios. 100 μl of IgA conjugate (Goat Antihuman IgA Peroxidase 1:10,000 concentration using a blocking buffer) was added to each well and incubated for 1 h at room temperature. 100 μl of sulfuric acid (H_2_SO_4_) stop solution was then added to stop the reaction and the optical density taken at 450 nm into iMARK microplate reader. For the assays, the antigens contained a mixture of strains representing six serotypes of *A. actinomycetemcomitans*. The strains were (a) ATCC 29523, (b) ATCC 43718, (c) ATCC 33384, (d) IDH 781, (e) IDH 1705, and (f) C59A [21, 22]. The final levels of pathogen-specific immunoglobulins were normalized in accordance with the serum samples reference and were expressed in ELISA units (EU). The coefficient of interassay variation was 4.1% for serum *A. actinomycetemcomitans* IgG and 4.2% for *A. actinomycetemcomitans* IgA, as previously reported [22].

### Sample size analysis

The sample size analysis was determined before the study by considering two groups of patients, an effect size of 0.27 for serum *A. actinomycetemcomitans* IgG (primary outcome chosen), an expected standard deviation of 0.5 [20], a two-sided significance of 0.05 and a power of 80%. It was established that at least 44 subjects per single group were required for a good power sample level. In each group around 48 people were finally enrolled, so that a power value of 81% was obtained.

### Statistical analysis

For the statistical analysis, numerical data is represented as median and interquartile range or mean ± standard deviation (SD), and number and percentage for categorical variables. For data, a non-parametric approach was chosen because most examined variables did not have a normal distribution, such as that verified by the Kolmogorov Smirnov test. In order to compare the numerical data in the two groups, the Mann-Whitney test was applied.

The non-parametric Spearman correlation test was applied in order to establish any significant interdependence between *A. actinomycetemcomitans* IgG and periodontal parameters analyzed in all enrolled patients and then for a single group. The same test was used to evaluate significant interdependence between serum and salivary *A. actinomycetemcomitans* IgG versus hs-CRP. Furthermore, to analyze the association among diabetes (expressed such as yes/no) and *A. actinomycetemcomitans* IgG, a biserial point correlation was used.

Periodontal parameters are represented with mean ± SD and the comparisons between groups (periodontitis and controls) with the relative *p*-value. In order to assess whether periodontal parameters were influenced (increasing or decreasing) with a *A. actinomycetemcomitans* IgG increase, p-trend quartiles of *A. actinomycetemcomitans* IgG levels were obtained, and for each quartile of *A. actinomycetemcomitans* IgG, a mean and standard deviation (±SD) of all periodontal parameters was calculated. The Jonckheere-Terpstra Test was applied for the main analyzed periodontal variables to evaluate a p-trend for ordered quartiles of *A. actinomycetemcomitans* IgG. In particular, the Jonckheere-Terpstra Test was performed to determine whether serum and salivary *A. actinomycetemcomitans* IgG were statistically increased in the analyzed groups.

A stepwise multivariable linear regression model was used in order to analyze the dependence of every single periodontal parameter by explicable variables as gender, age, BMI, serum and salivary *A. actinomycetemcomitans* IgA, *A. actinomycetemcomitans* IgG, hs-CRP, high-density lipoprotein (HDL) and low-density lipoprotein (LDL) cholesterol.

Moreover, univariate and multivariable linear regression analyses were performed to evaluate the dependence of serum *A. actinomycetemcomitans* IgG levels (which were normally distributed) by possible predictors including age, sex, educational level, socioeconomic status (SES), smoking (yes/no), triglycerides, LDL cholesterol, HDL cholesterol, and taking into account possible confounders such as periodontitis, BMI and hs-CRP. The same regression analysis was performed for salivary *A. actinomycetemcomitans* IgG levels. Among possible predictors, serum *A. actinomycetemcomitans* IgG levels was also added. A two-sided *P*-value < 0.05 was considered to be statistically significant. All statistical analyses were made using a statistical software program (SPSS 22.0 for the Windows package; SPS Srl, Bologna, Italy).

## Results

### Patients characteristics

Demographic characteristics of the enrolled patients are represented in Table 1. All patients were Caucasians and were well matched for age (*p* = 0.076), gender (*p* = 0.149), BMI (*p* = 0.082) and number of smoking subjects (*p* = 0.119) (Table 1). PT patients presented higher median hs-CRP values [0.58 (0.37–0.69) mg/dl] in comparison with healthy controls [0.36 (0.28–0.41) mg/dl] (*p* < 0.001) (Table 1).

**Table 1:** Descriptive statistics of examined groups and comparison among them. Values are represented such as number and percentage or median and interquartile range (IQR) (1st; 3rd)

CLINICAL FEATURES	Reference Values	Healthy Controls (***n*** = 48)	Periodontitis (***n*** = 53)	***P***-value
Male, n. (%)		23 (48)	26 (49)	0.149
Age, mean ± SD		49.4 ± 4.6	50.8 ± 3.2	0.076
*Education level*				
Primary school, n (%)		16 (33.3)	18 (34)	0.231
High school, n (%)		17 (35.4)	19 (35.8)	0.335
College/University, n (%)		15 (31.2)	16 (30.2)	0.089
BMI, Kg/m^2^, mean ± SD		23.7 ± 2.46	24.1 ± 2.39	0.082
*Smoker, n. (%)*		3 (6.2)	4 (7.5)	0.119
Current, n. (%)		1 (2)	2 (3.8)	0.119
Past, n. (%)		2 (4.1)	2 (3.8)	–
Never, n. (%)		45 (93.7)	49 (92.4)	0.204
Glucose, mg/dl	65–110	115.6 (89.5; 123.4)	119.6 (95.2; 133.4)	0.324
HbA1c, mmol/mol	up to 40	36.8 (32.3; 38.7)	37.6 (30.1; 47.6)	0.101
Uric acid, mg/dl	1.9–8	2 (1.5; 2.9)	2.3 (1.8; 3.1)	0.145
Albumin, g/L	35–50	38.6 (31.1; 42.4)	39.5 (33.2; 41.4)	0.198
Fibrinogen, mg/dl	150–400	278.5 (223.5; 295.6)	288.7 (253.2; 321.2)	0.047
Apolipoprotein A, mg/dl	> 120–140	134.2 (123.5; 132.5)	136.5 (129.3; 143.2)	0.442
Total cholesterol, mg/dl	< 200	177.6 (166.4: 184.4)	180.4 (158.4; 198.4)	0.456
HDL-cholesterol, mg/dl	< 40–60	48.5 (47.9; 59.4)	53.8 (47.4; 59.7)	0.511
LDL-Cholesterol mg/dl	< 100–130	115.8 (109.8; 128.8)	119.8 (108.2; 126.5)	0.532
BUN, mg/dl	7–30	26.7 (24.6; 29.8)	28.7 (25.6; 33.8)	0.210
hs-CRP, mg/dl	< 0.8	0.36 (0.28; 0.41)	0.58 (0.37; 0.69)	< 0.001
Systolic pressure, mm/hg	110–130	117.4 (111.7; 128.2)	123.6 (117.6; 133.2)	0.359
Diastolic pressure, mm/hg	70–85	81.9 (74.5; 85.6)	83.6 (76.4; 85.8)	0.111
Ferritin, ng/ml	12–300	77.6 (72.4; 83.9)	81.4 (74.5; 86.1)	0.786
Serum antibody levels, EU				
*A. actinomycetemcomitans* IgA		1.37 (0.9–1.8)	1.89 (1.2–2.2)	0.022
*A. actinomycetemcomitans* IgG		2.18 (1.8–2.1)	2.96 (2.1–3.7)	< 0.001
Salivary antibody levels, EU				
*A. actinomycetemcomitans* IgA		1.42 (0.9–1.6)	1.67 (1.4–2.1)	0.019
*A. actinomycetemcomitans* IgG		1.84 (1.4–2)	2.19 (1.8–2.5)	0.028

Analysis of periodontal parameters comparisons is represented in Table 2. PT patients had a significantly lower number of teeth and higher levels of PD, CAL and BOP compared to healthy subjects (p < 0.001), while the PI score was similar between groups (*p* = 0.108).

**Table 2:** Descriptive statistics of periodontal parameters of examined groups and comparison among them. Values are represented, such as median and interquartile range (IQR) (1st; 3rd)

PERIODONTAL INDEXES	Controls (n = 48)	Periodontitis (n = 53)	P-value
Number of teeth, n.	23.8 (19–27)	18.9 (16–23)	< 0.001
CAL, mm	1.39 (1.21–1.76)	3.78 (3.5–4.1)	< 0.001
Sites with CAL 4–5 mm, %	–	38.6 (36.5–41.2)	–
Sites with CAL ≥ 6 mm, %	–	24.5 (22.6–26.1)	–
PD, mm	1.58 (1.21–2.24)	4.23 (3.55–4.87)	< 0.001
Sites with PD 4–5 mm, %	–	41.2 (38.1–43.2)	–
Sites with PD ≥ 6 mm, %	–	26.8 (23.4–28.7)	–
BOP, %	13.2 (9.1–17.7)	44.1 (38.2–47.4)	< 0.001
Plaque Index (PI), score	0.65 (0.51–0.72)	0.71 (0.63–0.83)	0.108

### Primary outcomes results

Compared to HC, patients with PT had significantly higher IgA [serum: PT, 1.89 (1.2–2.2) EU vs HC, 1.37 (0.9–1.8) EU (*p* = 0.022); saliva: PT, 1.67 (1.4–2.1) EU vs HC, 1.42 (0.9–1.6) EU (*p* = 0.019)] and *A. actinomycetemcomitans* IgG levels [serum: PT, 2.96 (2.1–3.7) EU vs HC, 2.18 (1.8–2.1) EU (*p* < 0.001); saliva, PT, 2.19 (1.8–2.5) EU vs HC, 1.84 (1.4–2) EU (*p* = 0.028)] (Table 1).

No significant association was found between serum and salivary *A. actinomycetemcomitans* IgG levels (r_s_ = 0.198, *p* = 0.294).

**Fig. 2:**
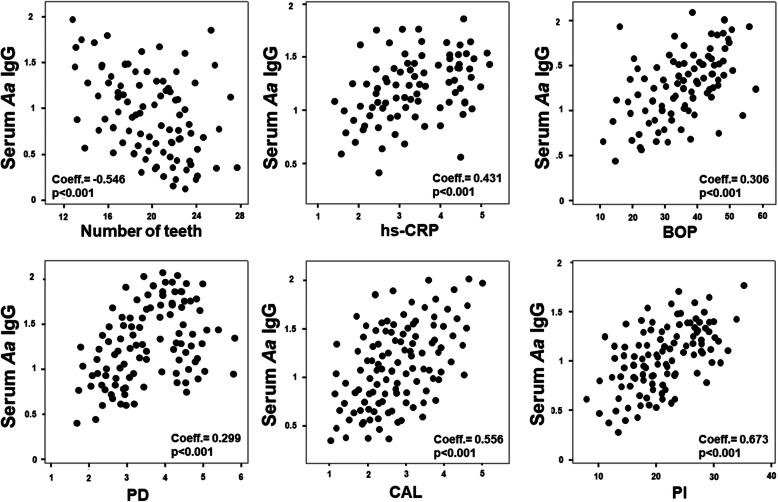
Spearman Correlation among serum *A. actinomycetemcomitans* IgG, hs-CRP and periodontal parameters

**Fig. 3:**
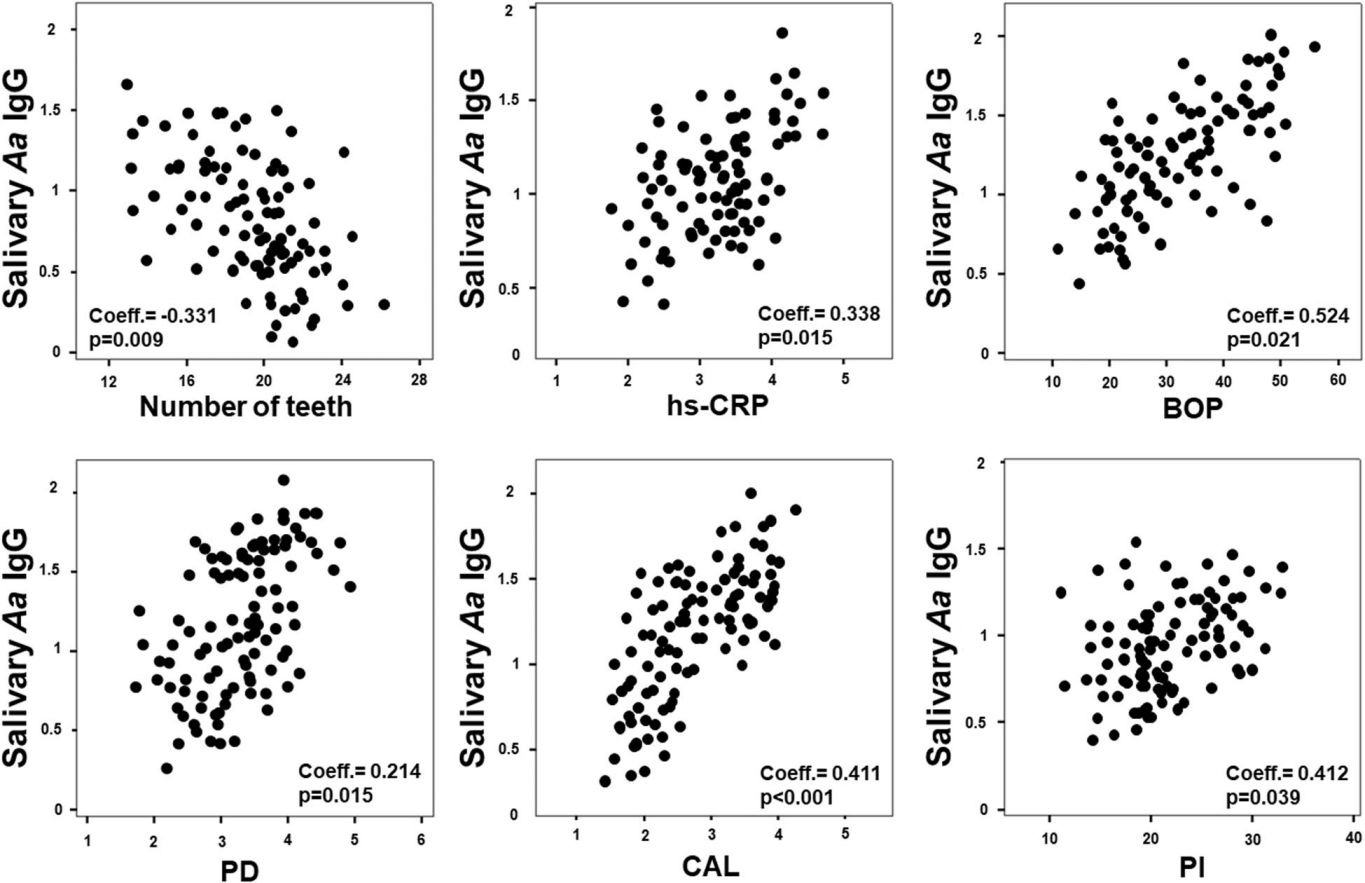
Spearman Correlation among salivary *A. actinomycetemcomitans* IgG, hs-CRP and periodontal parameters

The Spearman correlation analysis highlighted that, in all patients, serum *A. actinomycetemcomitans* IgG levels presented a negative correlation with the number of teeth (coeff. = − 0.546, *p* < 0.001) and a positive correlation with high levels of hs-CRP (coeff. = 0.431, p < 0.001), BOP (coeff. = 0.306, p < 0.001), PD (coeff. = 0.299, p < 0.001), CAL (coeff. = 0.556, p < 0.001), and PI (coeff. 0.673, p < 0.001) (Fig. 2). Regarding salivary *A. actinomycetemcomitans* IgG, there was a negative correlation with the number of teeth (coeff. = − 0.331, *p* = 0.009) and a positive correlation with high levels of hs-CRP (coeff. = 0.338, *p* = 0.015), BOP (coeff. = 0.254, *p* = 0.021), PD (coeff. = 0.214, p = 0.015), CAL (coeff. = 0.411, p < 0.001), and PI (coeff. 0.412, *p* = 0.039) (Fig. 3).

**Fig. 4:**
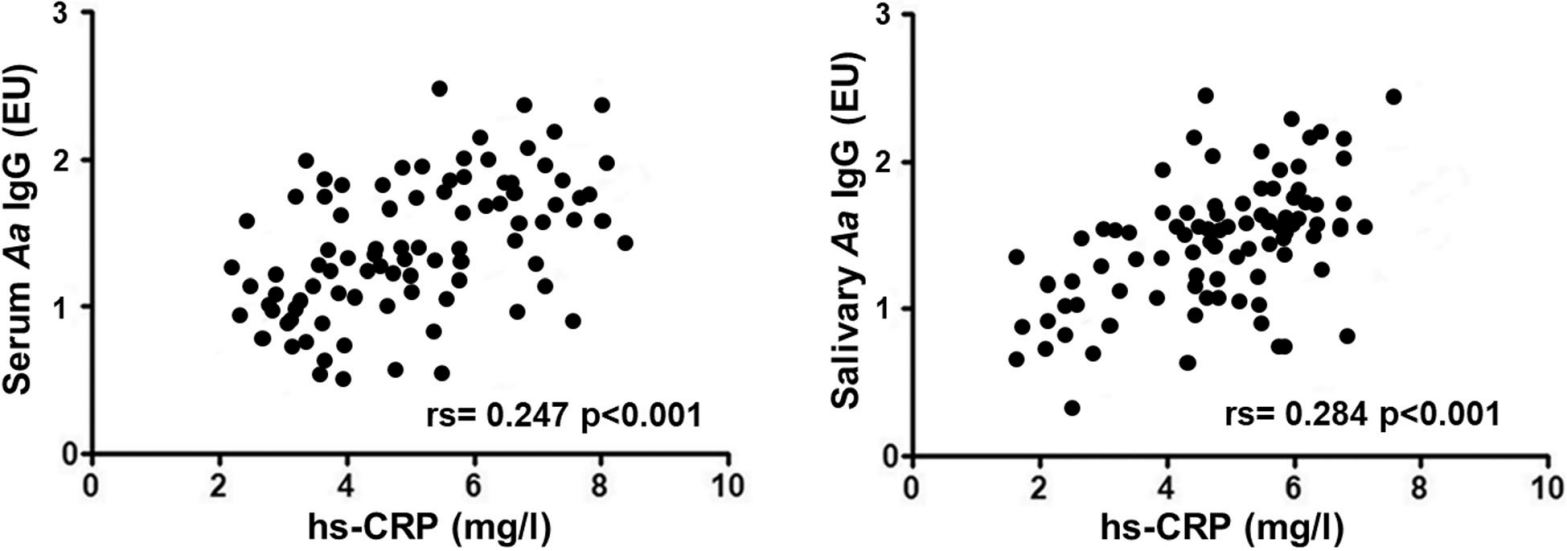
Correlation analysis of serum and salivary *A. actinomycetemcomitans* IgG levels with hs-CRP

Moreover, hs-CRP resulted positively correlated with serum (r_s_ = 0.247, p < 0.001) and salivary *A. actinomycetemcomitans* IgG (r_s_ = 0.284, p < 0.001) (Fig. 4).

**Fig. 5:**
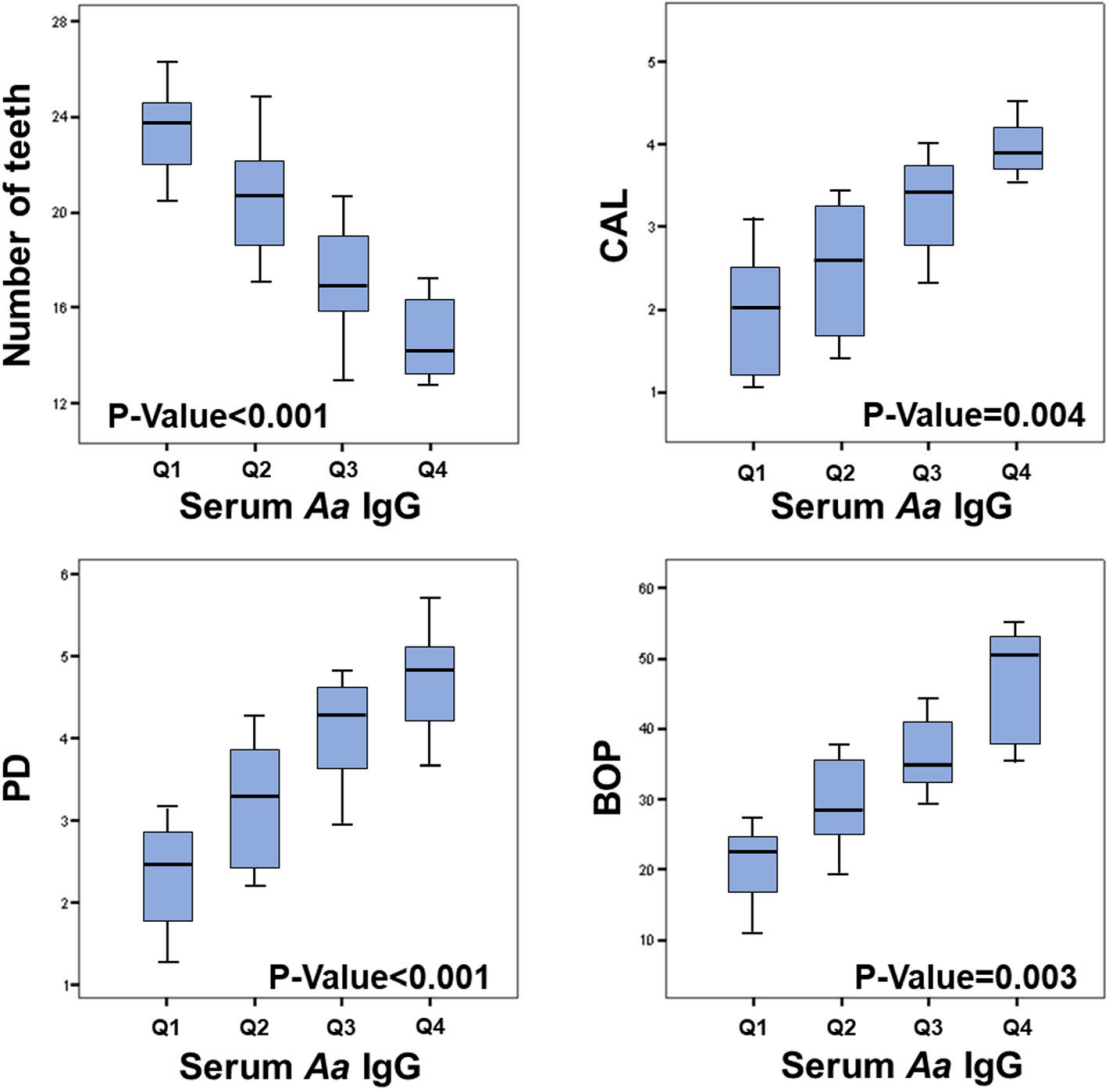
Analysis of periodontal parameters according to *A. actinomycetemcomitans* IgG quartiles and P-trend. Each P-trend value, referred to an increase/decrease of periodontal parameters according to *A. actinomycetemcomitans* IgG quartiles and was obtained by means of the Jonckheere-Terpstra test for the ordered alternative hypothesis

The p-trend obtained with the Jonckheere-Terpstra test showed that there was a significant decrease in the number of teeth in the four quartiles of serum *A. actinomycetemcomitans* IgG (P-trend< 0.001), while there was a proportional increase in in CAL (P-trend = 0.004), PD (P-trend< 0.001), and BOP (P-trend = 0.003) (Fig. 5), while there was no significant p-trend for PI (P-trend = 0.089). The Jonckheere-Terpstra test, instead, showed that there was no significant p-trend in quartiles of salivary *A. actinomycetemcomitans* IgG, for the number of teeth (P-trend = 0.189) and for any periodontal parameter analyzed, such as PD (P-trend = 0.251), CAL (P-trend = 0.339), BOP (P-trend = 0.556) and PI (P-trend = 0.412) (data not shown).

The stepwise multivariate regression analysis highlighted that the number of teeth, CAL, PD, and BOP were significantly dependent on serum *A. actinomycetemcomitans* IgG (*p* < 0.001 for all parameters) (Table 3).

**Table 3:** Stepwise (backward elimination) linear regression models for periodontal parameters. Only significantly predictors in the final models were reported

	**Number of teeth**			**CAL**		
**VARIABLES**	**Coeff.**	**95%C.I.**	**P-value**	**Coeff.**	**95%C.I.**	**P-value**
Age	− 0.25	− 0.22; 0.06	0.029	0.14	0.02; 0.57	0.034
Serum *A.actinomycetemcomitans* IgG	−0.27	− 0.11; 0.51	< 0.001	0.24	− 0.05; 0.83	< 0.001
hs-CRP	−1.66	−1.15; −1.19	0.003	0.43	0.41; 0.76	< 0.001
	**PD**			**BOP**		
**VARIABLES**	**Coeff.**	**95%C.I.**	**P-value**	**Coeff.**	**95%C.I.**	**P-value**
Age	−0.28	−0.12; 0.34	0.075	0.24	0.11; 1.78	0.003
Serum *A.actinomycetemcomitans* IgG	0.16	0.11; 0.76	< 0.001	1.44	1.29; 3.12	< 0.001
hs-CRP	0.51	0.25; 0.87	< 0.001	5.13	3.97; 6.81	< 0.001

More specifically, the number of teeth was significantly dependent also on age (*p* = 0.029) and hs-CRP (*p* = 0.003); CAL was also significantly dependent on age (*p* = 0.034) and hs-CRP (*p* < 0.001). PD was significantly dependent on hs-CRP (p < 0.001); BOP was significantly dependent on age (p = 0.003), hs-CRP (p < 0.001) (Table 3). The other considered confounders were not significant (data not shown).

Finally, the univariate regression analysis demonstrated that PT (*p* = 0.013), high hs-CRP (p < 0.001) and BMI (p < 0.001) had a significant negative effect on serum *A. actinomycetemcomitans* IgG. The same predictors were found to be significant for salivary *A.actinomycetemcomitans* IgG (PT, *p* = 0.019; hs-CRP, p < 0.001; BMI, *p* = 0.036).

The multivariate regression analysis showed that PT (*p* = 0.033), hs-CRP (*p* = 0.014) and BMI (*p* = 0.017) were significant predictors of serum *A.actinomycetemcomitans* IgG while hs-CRP (p < 0.001) and BMI (*p* = 0.025) were the significant predictors of salivary *A. actinomycetemcomitans* IgG (Table 4).

**Table 4:** Uni and multivariate linear regression analysis for serum and salivary *A.actinomycetemcomitans* IgG in all patients. Age was included as continuous variable. For PT, controls served as reference. For gender, male served as reference. SES, socioeconomic status; BMI, PT, periodontitis

	Variable	Univariate model			Multivariate model		
		B	95% CI	***P***	B	95% CI	***P***
**Serum** ***A. actinomycetemcomitans*** **IgG levels**	**Age (years)**	−0.045	−0.125;0.155	0.236	− 0.112	− 0.118; 0.412	0.554
	**Male gender**	0.312	−0.101;0.419	0.345	0.327	−0.332; 0.412	0.055
	**Education**	−0.041	−0.198;0.289	0.187	−0.085	− 0.318; 0.289	0.498
	**SES**	0.185	−0.122;0.296	0.396	0.219	−0.231;0.155	0.254
	**Smoking**	0.174	−0.029;0.321	0.258	0.156	−0.147;0.187	0.199
	**Triglycerides**	0.136	−0.111;0.365	0.221	0.129	−0.105;0.321	0.187
	**LDL Cholesterol**	0.158	0.096;0.254	0.155	0.115	−0.123;0.221	0.069
	**HDL Cholesterol**	0.141	0.116;0.365	0.134	0.108	−0.085;0.344	0.075
	**PT**	0.298	0.212;0.478	0.013	0.315	−0.041;0.458	0.033
	**hs-CRP**	0.419	0.029;0.652	< 0.001	0.374	0.157;0.336	0.014
	**BMI**	0.321	0.96;0.441	< 0.001	0.254	0.125;0.452	0.017
**Salivary** ***A. actinomycetemcomitans*** **IgG levels**	**Age (years)**	−0.112	−0.021;0.112	0.335	0.048	−0.047;0.178	0.551
	**Male gender**	0.187	−0.155;0.289	0.214	0.098	−0.058;0.410	0.554
	**Education**	−0.145	−0.189;0.113	0.412	−0.074	− 0.023;0.201	0.328
	**SES**	0.211	−0.099;158	0.321	0.055	0.022;0.211	0.352
	**Smoking**	0.158	−0.254;0.078	0.258	0.147	−0.052;0.256	0.214
	**Triglycerides**	0.289	−0.187;0.554	0.654	0.369	−0.056;0.441	0.551
	**LDL Cholesterol**	0.199	−0.025;0.325	0.294	0.211	0.058;0.351	0.234
	**HDL Cholesterol**	0.174	−0.078;0.365	0.551	0.089	0.027;0.169	0.337
	**PT**	0.158	−0.045;0.336	0.019	0.033	−0.112;0.245	0.141
	**hs-CRP**	0.096	0.045;0.214	< 0.001	0.047	0.039;0.158	< 0.001
	**BMI**	0.113	0.058;0.199	0.036	0.058	0.078;0.201	0.025
	**Serum** ***A. actinomycetemcomitans*** **IgG levels**	0.141	−0.117;0.421	0.189	−0.116	−0.147;0.278	0.258

## Discussion

The present study analyzed the association between serum and salivary IgG antibodies against *A. actinomycetemcomitans* and PT. Furthermore, was evaluated the impact of PT and gingival health on serum and salivary levels of *A. actinomycetemcomitans* IgG, as these were the main confounders that could determine a significant influence in healthy subjects and PT patients. The results highlighted that patients with PT presented significantly higher serum and salivary *A. actinomycetemcomitans* IgG in comparison to healthy subjects.

In agreement with our results, previous studies showed that patients with PT had significantly higher serum *A. actinomycetemcomitans* IgG than healthy patients [23, 24].

In this regard, some reports showed the central role of *A. actinomycetemcomitans*, together with some others periodontal pathogens, in the etiology and progression of PT [25] and to be more prevalent in active sites with PT compared with non-diseased sites in the same patients [26]. *A. actinomycetemcomitans* has been identified between the major periodontal pathogens involved in the juvenile forms of PT [27] and decrease slightly with advancing age [15, 28]. However, recent evidences has shown a bacterial burden and prevalence of *A. actinomycetemcomitans* in the onset of aggressive and adult forms of PT, whereas the subgingival microbiota tends to shift toward red-complex pathogens as the disease progresses and related inflammatory response [20, 29–31].

Furthermore, it has also been demonstrated that *A. actinomycetemcomitans* strains could predict, in the early stages, the appearance of PT and that serum titer Ig antibodies against *A. actinomycetemcomitans* could be helpful in evaluating the degree of PT, with a greater possibility to develop tissue destruction compared to periodontal sites which don’t harbour *A. actinomycetemcomitans* [32] since they remain remarkably stable over a long period of time [15, 33].

In our study, serum and salivary IgG antibodies against *A. actinomycetemcomitans* were correlated with the extent and degree of the disease in PT patients (PD, CAL, BOP and the number of teeth). In accordance, recent evidence has shown that serum IgG against *A. actinomycetemcomitans* could also have a role in the pathogenesis of PT through a specific pathway in association with different cytokines that finally determines the destruction of collagen in periodontal tissues finally [31].

Recent evidence introduced the hypothesis that high IgG antibodies against *A. actinomycetemcomitans* could lead to an increase in the autoimmune and inflammatory systemic response, especially during PT [20, 34, 35]. Previous studies, carried out in both animal and human models, have shown that high serum IgG *A. actinomycetemcomitans* antibodies levels are associated to a higher risk of developing alveolar bone and tooth loss [9, 15, 36, 37]. In this regard, it is reasonable to speculate that analysis of serum IgG antibodies could be valid markers for exposure to periodontal pathogens in the long-term period [15, 33]. Specifically, Lakio et al. have shown that serum *P. gingivalis* IgG antibodies level and *A. actinomycetemcomitans* IgG antibody level were particularly stable over fifteen years in patients with PT, associated with the extent of PT [16]. In accordance, the present study showed that, in enrolled patients, the number of teeth and the levels of PD, CAL and BOP were significantly dependent on serum *A. actinomycetemcomitans* IgG antibody levels (*p* < 0.001 for all observations). These results were in agreement with previous pivotal observations which highlighted a possible relationship between PT and high *A. actinomycetemcomitans* levels in gingival biofilm [38].

Therefore, based on these preliminary observations, the present study was aimed at further analyzing the relationship between serum and salivary *A. actinomycetemcomitans* IgG levels and PT and the effects exerted by PT and gingival health on serum and salivary *A. actinomycetemcomitans* IgG levels.

In light of this, interestingly, the results of the present study showed that, in the analyzed sample, there was a significant decrease in the number of teeth in the four quartiles of serum A. actinomycetemcomitans IgG, while there was a significant proportional increase in CAL, PD, and BOP. Moreover, the multivariate regression analysis showed that PT, hs-CRP and BMI were significant predictors of serum *A.actinomycetemcomitans* IgG while hs-CRP and BMI were the significant predictors of salivary *A. actinomycetemcomitans* IgG, confirming the hypothesis that PT may have a negative influence on serum and salivary *A. actinomycetemcomitans* IgG levels. In agreement, previous researchers have shown that in patients who had high serotype frequency antibody titers of *A. actinomycetemcomitans* IgG (from 28 to 23%) have a net risk of developing PT [39]. Furthermore, other reports have highlighted that patients with optimal low levels of serum *A. actinomycetemcomitans* IgG and IgA titers may represent a good chanced having better gingival health conditions associated with low levels of interleukin (IL)-1β and IL-10, tissue-destruction mediators during PT and coronary diseases [40–43].

Furthermore, the present study also suggested that serum hs-CRP were positively correlated with serum and salivary *A. actinomycetemcomitans* IgG levels and also that, together with PT and BMI, was a significant negative predictor of both serum and salivary *A. actinomycetemcomitans* IgG.

Some of the major issues associated with PT were the tissue hypoxia, oxidative stress and relative vasospasm which in turn, determines increased coagulation and endothelial injury, damage of vascular endothelial cells, and finally periodontal tissue destruction [44]. Consequently, some evidence has been suggested that periodontal infection mediated by some pathogens such as *A. actinomycetemcomitans* may accelerate PT and endothelial damage and inflammatory response orchestrated through oxidative stress pathways [45, 46], especially in obese patients [47]. In this respect, some evidence has shown that the tissue damage associated with PT was due to oxidative stress and, therefore, associated with high levels of nitric oxide (NO) and hs-CRP [48]. The high production and release of both oral and salivary of both hs-CRP and NO can be deduced from the demonstrated evidence that NO is released orally following the host’s specific defence immune response on the infection triggered by periodontal pathogens that are exacerbated during PT [49, 50] and that could led to an increase of local and systemic *A. actinomycetemcomitans* IgG levels.

Moreover, it has been previously underlined the characteristics of *A. actinomycetemcomitans* IgG titers in the influence the inflammatory response, have previously been underlined and may interfere on the systemic release of hs-CRP [51–53]. On another hand, the association between *A. actinomycetemcomitans* IgG antibodies, hs-CRP and PT could be explained by a strong influence exerted by PT on systemic inflammation, which could determine a further release of hs-CRP systemically [54]. In accordance with this hypothesis, previous studies [55–57], have demonstrated that hs-CRP levels were negatively correlated, in a dose-dependent manner, with periodontal inflammation in patients with PT, even after adjustment for potential confounders [58]. On this regard the presence of PT has been demonstrate to determine a further systemic inflammation and augmented risk of endothelial dysfunction [10, 59–61].

The present study has some limitations, such as the study design. The cross-sectional nature of the study design does not allow to a better analysis of the longitudinal relationship between PT and serum *A. actinomycetemcomitans* IgG titers. Moreover, staging the different severity levels of PT at baseline could have better-stratified patients.

## Conclusions

During the last few decades, a number of trials have tried to analyze the impact of IgG *A. actinomycetemcomitans* titers for human health and related diseases. The results of the present study indicated that PT patients presented higher serum and salivary *A. actinomycetemcomitans* IgG titers compared to healthy subjects. Moreover, PT and high hs-CRP were negative predictors of high levels of serum and salivary *A. actinomycetemcomitans* IgG titers. With regard to this, the results of this study suggest that PT may have led to a negative effect in serum and salivary *A. actinomycetemcomitans* IgG titers. However, further studies with a more large sample and different design are needed in order to analyze the role of *A. actinomycetemcomitans* IgG titers in PT patients better.

## Supplementary information

**Additional file 1.** STROBE Checklist.

## Data Availability

The datasets used and/or analysed during the current study available from the corresponding author on reasonable request.

## Abbreviations

Ig: Immunoglobulin
A: actinomycetemcomitans: Aggregatibacter actinomycetemcomitans
HC: healthy controls
PT: periodontitis
hs-CRP: high-sensitivity c-reactive protein
P. gingivalis: Porphyromonas gingivalis
BOP: bleeding on probing
PD: probing depth
CAL: clinical attachment level
BMI: body mass index
PI: plaque score
ICC: intraclass correlation coefficient
